# Hypoxia-induced LncRNA DACT3-AS1 upregulates PKM2 to promote metastasis in hepatocellular carcinoma through the HDAC2/FOXA3 pathway

**DOI:** 10.1038/s12276-022-00767-3

**Published:** 2022-06-28

**Authors:** Liyan Wang, Bin Li, Xiaotong Bo, Xiaoyuan Yi, Xuhua Xiao, Qinghua Zheng

**Affiliations:** 1 Digestive Department, the Affiliated Hospital of Guilin Medical College; Molecular Medicine of Liver Injury and Repair Collaborative Innovation Center, Guilin, 541001 Guangxi China; 2 Digestive Department, the Affiliated Hospital of Guilin Medical College, Guilin, 541001 Guangxi China

**Keywords:** Cancer, Cancer

## Abstract

Growing evidence has revealed that hypoxia is involved in multiple stages of cancer development. However, there are limited reports on the effects of long noncoding RNAs (lncRNAs) on hepatocellular carcinoma (HCC) progression under hypoxia. The main purposes of this study were to analyze the effect of the novel lncRNA DACT3-AS1 on metastasis in HCC and to elucidate the related molecular mechanism. Bioinformatics tools were employed. RT–qPCR or western blot assays were conducted to detect RNA or protein expression. Clinical samples and in vivo assays were utilized to reveal the role of DACT3-AS1 in HCC. Other mechanism and functional analyses were specifically designed and performed as well. Based on the collected data, this study revealed that HIF-1α transcriptionally activates DACT3-AS1 expression under hypoxia. DACT3-AS1 was verified to promote metastasis in HCC. Mechanistically, DACT3-AS1 promotes the interaction between HDAC2 and FOXA3 to stimulate FOXA3 deacetylation, which consequently downregulates the FOXA3 protein. Furthermore, FOXA3 serves as a transcription factor that can bind to the PKM2 promoter region, thus hindering PKM2 expression. To summarize, this study uncovered that HIF-1α-induced DACT3-AS1 promotes metastasis in HCC and can upregulate PKM2 via the HDAC2/FOXA3 pathway in HCC cells.

## Liver cancer: role of non-coding RNA in cancer spread

Understanding the role of an RNA molecule involved in metastasis (spread) of liver cancer may suggest potential therapeutic targets. Hepatocarcinoma is a common primary liver cancer, and mortality remains high due to late diagnosis and the risk of metastasis. Scientists believe hypoxic (low oxygen) conditions in solid tumors may trigger metastasis by a mechanism involving long non-coding RNAs. Bin Li and co-workers at the Affiliated Hospital of Guilin Medical College, China, used patient tissue samples to examine the role of the long non-coding RNA molecule DACT3-AS1 in promoting hepatocarcinoma metastasis. Hypoxia triggers the overexpression of HIF-1α. This protein activated DACT3-AS1, which was then highly expressed in metastatic tissues. DACT3-AS1 interacted with a nearby gene and associated enzyme to promote cell migration and invasion, hinting at possible treatment options.

## Introduction

Hepatocellular carcinoma (HCC) is the leading cause of cancer-related death worldwide^1^. Current mainstays of therapies for HCC include surgical resection, liver transplantation, tumor ablation, systemic chemotherapies, etc.^2^. It is noteworthy that only early-diagnosed HCC patients are eligible for curative treatments, such as radical surgery, ablation and liver transplantation, and have a favorable prognosis, with 5-year survival rates >70%. For patients at advanced stages of HCC, systemic chemotherapies are recommended. Nevertheless, the therapeutic effectiveness is unsatisfactory due to the chemoresistance of HCC cells^3,4^. Due to the lack of precise early diagnosis, the mortality of HCC remains high^5^. In addition, metastasis is a complicated, multistep biological process, implicating multiple genes and biomolecules, which is a critical characteristic of cancer that results in high fatality of cancer patients, particularly in HCC^6,7^. Therefore, uncovering the molecular mechanisms in HCC will facilitate the improvement of early HCC diagnosis precision and the development of efficient therapeutic targets.

Hypoxia represents an intrinsic trait of solid tumors, resulting from the imbalanced rate of tumor cell proliferation and nutrient supply to the vasculature, and the liver is an organ that is highly susceptible to hypoxia^8,9^. Emerging evidence has uncovered that hypoxia is closely associated with HCC development and poor prognosis, as hypoxic cancer cells are particularly aggressive, metastatic, and resistant to therapy^10,11^. In addition, hypoxia-mediated long noncoding RNAs (lncRNAs) have been demonstrated to induce tumor metastasis^12^. In recent years, increasing attention has been given to the relationship between hypoxia, lncRNAs and cancer. For instance, hypoxia-induced TUFT1 is proposed to facilitate HCC growth and metastasis by switching on the Ca(2+)/PI3K/AKT pathway^13^. Another study revealed that hypoxia-induced HMGB1 mediates HCC tumor growth via Toll-like receptor 9^14^. Jiang et al. recently proposed that HMGB1 induced by hypoxia boosts HCC tumor invasiveness and metastasis by modulating macrophage-derived IL-6^15^. According to previous reports, hypoxia-inducible factor 1 alpha (HIF-1α) mediates hypoxic responses and regulates the expression of genes involved in various cell processes^16^. Recent work elucidates that HIF-1α can upregulate VASP to promote invasiveness and metastasis of HCC^17^. In this study, we aimed to discover a novel lncRNA that was aberrantly expressed in hypoxic HCC cells and propose a new understanding of the potential regulatory mechanism of hypoxia-induced lncRNAs in HCC.

Recent research has proven that interaction between neighboring genes is a prevailing cellular activity that involves multiple mechanisms and cis-regulatory signals^18^. Some lncRNAs have been reported to affect the expression of chromosomally neighboring genes, thereby modulating the progression of cancers^19,20^. For example, lncRNA MAPKAPK5-AS1 was discovered to induce colorectal cancer development via cis-regulation of its nearby gene MK5 and functioning as a let-7f-1-3p sponge^21^. LncRNA SOX2-OT elevated by IRF4 facilitates cell proliferation and metastasis in cholangiocarcinoma by enhancing the expression of SOX2 and activating the PI3K/AKT signaling pathway^22^. Moreover, DARS-AS1 can positively modulate its nearby gene DARS by inhibiting miR-194-5p, thus promoting the progression of clear cell renal cell carcinoma^23^. Hence, the correlation between DACT3-AS1 and its nearby gene in HCC is worth exploring.

PKM2 is an enzyme that plays an important role in cancer cells, and it is elevated by hypoxia in liver cancer cell lines^24,25^. Furthermore, growing research has highlighted the critical role of hypoxia-increased PKM2 in cancer development. For instance, PKM2 downregulation results in weaker proliferative and invasive abilities of HCC cells under hypoxic conditions than under normoxic conditions^26^. In addition, PKM2 is upregulated in hypoxic HCC cells and can contribute to HCC development^27^. Therefore, PKM2 might also be a valuable subject in this study.

In conclusion, the present study primarily explores the role and mechanism of the novel lncRNA DACT3-AS1 in HCC cells under hypoxia, which is a relatively new topic in the current biomedical field. We hope this study will successfully identify potential targets for HCC treatment.

## Materials and methods

### Clinical specimens

Cancer tissues and paired adjacent noncancer tissues from 67 patients who underwent primary HCC surgery were acquired from the Affiliated Hospital of Guilin Medical College. All patients signed the informed consent. The study was approved by the Affiliated Hospital of Guilin Medical College. Among the patients, 38 patients had tumor metastasis, and 29 patients had tumors without metastasis. The expression level of DACT3-AS1 was investigated in fresh frozen HCC tissues and their corresponding adjacent normal tissues. All samples were immediately maintained in liquid nitrogen at −80 °C.

### Cell culture

Human HCC cells (SMMC-7721, Huh7, MHCC97H, SK-HEP-1, and HepG2) were used in this study. SMMC-7721 cells were obtained from the Chinese Academy of Sciences (CAS), Huh7 cells were acquired from the JCRB Cell Bank, MHCC97H cells were obtained from ATCC, and SK-HEP-1 cells together with HepG2 cells were procured from American Type Culture Collection (ATCC). SMMC-7721 cells were cultured in RPMI 1640 medium (61870127, Gibco, USA) supplemented with 10% fetal bovine serum (FBS; 10270-106, Gibco) and 1% penicillin–streptomycin solution (SV30010, PERFEMIKER, Shanghai, China). The remaining cells were grown in DMEM (12100046, Gibco) containing the same supplements mentioned above. All incubators were placed in a humidified environment with 5% CO_2_ at 37 °C. For the formation of hypoxic cells, HCC cells were maintained in an incubator containing 5% CO_2_, 1% O_2_ and 94% N_2_ or treated with 100 μM CoCl_2_ for 24–48 h.

### Cell transfection

HCC cells were seeded into six-well plates and incubated overnight. Then, cells were collected and transfected with different plasmids for RNA knockdown or overexpression. Short hairpin RNAs against HIF-1α, DACT3-AS1 or FOXA3 were constructed to downregulate RNAs. Meanwhile, pcDNA3.1 to overexpress HIF-1α, HDAC2, FOXA3 or PKM2 was also synthesized. The indicated plasmids along with the negative control (NC) were transfected into HCC cells by means of Lipofectamine 2000 (11668019, Invitrogen, USA).

### Quantitative reverse transcription polymerase chain reaction (RT–qPCR)

Total RNA extraction of HCC cells was achieved with TRIzol (9108-1, Thermo Fisher, USA). The obtained RNA was then reverse transcribed into complementary DNA (cDNA). Afterward, a qRT–PCR kit (QR0100-1KT, Sigma–Aldrich, USA) was employed to quantify the expression level of the RNAs. The 2^−∆∆Ct^ method was applied to measure the results after quantification. GAPDH was used as internal reference.

### Western blot

Protein lysis buffer (ZD409, ZOMANBIO, Beijing, China) and a Total Protein Extraction Kit (BC3711, Solarbio, Beijing, China) were employed to extract total proteins from HCC cells. The obtained proteins were isolated by SDS–PAGE (P1200, Solarbio) and then transferred onto PVDF membranes (T2234, Thermo Fisher). After blocking with 5% skimmed milk, the membranes were cocultured with primary antibodies, including anti-HIF-1α (ab1, Abcam), anti-β-actin (ab179467, Abcam), anti-MMP2 (ab92536, Abcam), anti-MMP9 (ab76003, Abcam), anti-ZO-1 (ab276131, Abcam), anti-E-cadherin (ab40772, Abcam), anti-N-cadherin (ab76011, Abcam), anti-vimentin (ab92547, Abcam), anti-Ac-K (ab190479, Abcam), anti-FOXA3 (PA1-813, Abcam), anti-HDAC2 (ab32117, Abcam), anti-PKM2 (ab137791, Abcam) and anti-DADACT3 (ab797, Abcam). Subsequently, the membranes were incubated with secondary antibody for 1 h. The protein levels were measured by means of an enhanced chemiluminescence detection system. β-actin served as the internal reference.

### Northern blot

Total RNA was isolated from HCC tissues and adjacent noncancer tissues using TRIzol. DACT3-AS1 and 18S rRNA probes for northern blotting were constructed. The RNA samples were separated by electrophoresis and then transferred onto nitrocellulose membranes. Later, hydration buffer containing these probes was added for incubation. Finally, RNA signal images were developed and analyzed.

### Wound healing assay

HCC cell migratory capacity was examined in wound healing assays. First, HCC cells transfected with different plasmids were cultured in 12-well plates. After the cells reached 90–100% confluence, a pipette tip was used to scrape off a row of cells above the plates. The wounded cells were then rinsed away with PBS (C10010500BT, Gibco). Later, the cells were incubated in serum-free DMEM. The distance of each wound was observed and measured under a microscope. Images were captured after cells were cultured for 0 and 24 h.

### Transwell assay

For the Transwell invasion assay, cells were suspended in the apical chamber with Matrigel-coated membrane containing serum-free medium. In addition, the basolateral camber was added with 10% FBS. After incubation, a cotton swab was used to wipe off the cells that did not traverse the membrane. The invaded cells were fixed and then stained with crystal violet. For the Transwell migration assay, the membrane of the apical chamber was not covered with Matrigel. The remaining procedures were the same as those for the Transwell invasion assay. The number of invaded or migrated cells was observed by light microscopy.

### RNA binding protein immunoprecipitation (RIP) assay

An Imprint^®^ RNA Immunoprecipitation Kit (RIP-12RXN, Sigma–Aldrich, USA) was utilized to perform the RIP assay. Manufacturer’s instructions were strictly followed. Cell lysates were incubated with NC anti-immunoglobulin G (IgG) (ab6789, Abcam, USA), anti-FOXA3 or anti-HDAC2 conjugated with magnetic beads. After purification, the immunoprecipitated RNAs were isolated, and quantitative data were obtained by RT–qPCR analyses.

### Luciferase reporter assay

The wild-type (WT) or Site1-mutated (Site1 Mut) sequence of the DACT3-AS1 promoter was cloned into pGL3 reporter vectors to construct the pGL3-DACT3-AS1 promoter (WT) and pGL3-DACT3-AS1 promoter (Site1 Mut). Likewise, the pGL3-PKM2 promoter (WT), pGL3-PKM2 promoter (Site1 Mut) and pGL3-PKM2 promoter (Site2 Mut) were synthesized. After plasmid transfection, a Dual Luciferase Reporter Assay Kit (DL101-01, Vazyme, Nanjing, China) was utilized to examine the luciferase activities, following the supplier’s manual. After transfection for 48 h, the relative luciferase activities were detected and normalized to Renilla reniformis activity.

### RNA pull-down assay

Biotinylated (Bio) DACT3-AS1 sense/anti-sense was designed and synthesized with the application of the Pierce^™^ RNA 3′ End Desthiobiotinylation Kit (20160, Thermo Fisher) according to the supplier’s instructions. Then, biotin-labeled probes were incubated with cell lysates and magnetic beads overnight. After purification, enriched proteins were subjected to western blot analysis.

### DNA pull-down assay

A Pierce^™^ Biotin 3′ End DNA Labeling Kit (89818, Thermo Fisher) was employed to produce the Bio-DACT3-AS1/PKM2 promoter and Bio-NC probes. Afterward, a DNA Pull-down Kit (KT104-01, gzscbio) was utilized to evaluate the binding of DNA and protein according to the manufacturer’s guidelines. The protein pulled down was analyzed via western blot.

### Chromatin immunoprecipitation (ChIP)

A ChIP Kit (KT101-01, gzscbio, Guangzhou, China) was purchased to perform the ChIP assay. Briefly, crosslinked chromatin was sliced into small sections. Then, anti-IgG/anti-HIF-1α/anti-FOXA3 antibodies as well as magnetic beads were added for cocultivation. Finally, qPCR was conducted to detect the expression of enriched DNA.

### Glutathione-S-transferase (GST) pull-down assay

GST pull-down assays were performed to analyze protein–protein interactions. GST fusion probe protein was added to the cell lysates and incubated at 4 °C for 2 h. GST-tagged proteins were isolated by means of glutathione-coated beads. After elution, the obtained protein complexes were subjected to SDS–PAGE and further analyzed via mass spectrometry.

### Xenograft assay

Male BALB/c nude mice (6–8 weeks old) were purchased from Guangdong Medical Laboratory Animal Center. These mice were randomly divided into two groups. SMMC-7721 cells were transfected with sh-NC or sh1-DACT3-AS1 plasmids and then incubated for 48 h. Afterward, 2 × 10^6^ SMMC-7721 cells with stable transfection were subcutaneously injected into each mouse. The tumor volume was first monitored on the 7th day and monitored every 3 days thereafter (formula: length × width^2^/2). Twenty-eight days later, tumors were isolated from the sacrificed mice, and the weight was measured. Animal experiments were approved by the Affiliated Hospital of Guilin Medical College.

### Immunohistochemistry (IHC)

In this study, an IHC assay was used to analyze the expression of key proteins related to cell proliferation, neoangiogenesis and epithelial-mesenchymal transition (EMT) in xenograft tumor tissues. First, the obtained tissues were fixed with 4% PFA and embedded in paraffin. Then, the samples were sectioned into tiny slices and mounted on the slides. Later, these slices were blocked and incubated with primary antibodies targeting Ki-67 (a cell proliferation marker), CD31 (a neoangiogenesis and stem cell marker), E-cadherin (an EMT negatively related protein) and Vimentin (an EMT positively related protein) at 4 °C overnight. After being washed with PBS, the sections were cocultured with secondary antibodies for an hour. Finally, the washed and dehydrated sections were stained with DAB substrate and observed under a fluorescence microscope.

### Statistical analysis

The SPSS statistical software package and GraphPad Prism were used for statistical analysis. Each independent experiment was implemented at least three times. The obtained data are presented as the mean ± standard deviation (SD). Statistical differences between groups were analyzed by Student’s *t* test, one-way analysis of variance (ANOVA) or two-way ANOVA. A *P* < 0.05 was deemed to indicate statistical significance.

## Results

### HIF-1α transcriptionally activates DACT3-AS1 expression under hypoxia

**Fig. 1:**
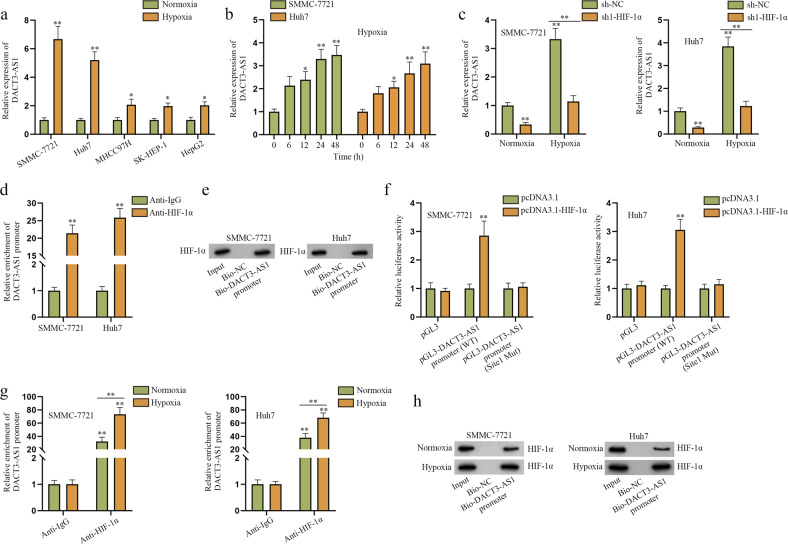
HIF-1α promotes transcription of DACT3-AS1 in hypoxic cells. **a** RT–qPCR was performed to detect the expression of DACT3-AS1 in HCC cells under normoxia and hypoxia. **b** RT–qPCR was conducted to determine the expression of DACT3-AS1 in hypoxic SMMC-7721 and Huh7 cells every 6 h. **c** The expression of DACT3-AS1 was tested via RT–qPCR in normoxic and hypoxic HCC cells transfected with sh1-HIF-1α. **d**–**e** ChIP and DNA pull-down assays were carried out to evaluate the interaction between the DACT3-AS1 promoter and HIF-1α. **f** The binding sites of HIF-1α on the DACT3-AS1 promoter were verified with the help of a luciferase reporter assay. **g**–**h** The binding ability between the DACT3-AS1 promoter and HIF-1α was confirmed in HCC cells under normoxia and hypoxia by utilizing ChIP and DNA pull-down assays. *P < 0.05, **P < 0.01.

According to emerging studies, hypoxia impacts the development of HCC and can induce the aberrant expression of lncRNAs^8,28^. To reveal the role of lncRNA DACT3-AS1 in HCC cells under hypoxia, a series of experiments were designed. In the majority of the following experiments, HCC cells were incubated under hypoxia (1% O_2_, 5% CO_2_ and 94% N_2)_ for 24–48 h, except for the kinetic study. RT–qPCR analysis was conducted to detect the expression of DACT3-AS1 in HCC cells under normoxia/hypoxia. As shown in Fig. 1a, hypoxic SMMC-7721 and Huh7 cells had remarkably high expression of DACT3-AS1, and therefore, these cell lines were selected for the following experiments. Furthermore, we detected DACT3-AS1 expression at regular intervals in HCC cells under hypoxia. The RT–qPCR results showed that DACT3-AS1 expression increased with time (Fig. 1b). Recently, growing evidence has revealed that HIF-1α participates in multiple processes of hypoxic tumor cells and acts as an essential transcription factor induced by hypoxia^29,30^. Based on RT–qPCR and western blot analyses, the RNA and protein levels of HIF-1α were determined, and the data showed that HIF-1α was highly expressed in HCC cells after 48 h of hypoxia treatment compared with normoxic HCC cells (Supplementary Fig. 1a). Prior to exploring the effect of HIF-1α on DACT3-AS1 expression, we confirmed the effective knockdown of HIF-1α in HCC cells (Supplementary Fig. 1b). The following RT–qPCR data illustrated that hypoxia treatment led to an increase in DACT3-AS1 expression, while downregulation of HIF-1α hindered this induction, which suggested that the expression of DACT3-AS1 was regulated by HIF-1α in hypoxic HCC cells (Fig. 1c). Subsequently, ChIP and DNA pull-down assays were performed to determine whether the transcription factor HIF-1α could bind to the DACT3-AS1 promoter region. The results shown in Fig. 1d–e firmly supported their binding affinity and indicated that HIF-1α functioned as the transcription factor for DACT3-AS1. Based on the prediction of binding sites on the JASPAR (http://jaspar.genereg.net/) database (Supplementary Fig. 1c), we designed luciferase reporter assays. Only the first predicted sequence was involved because its score was more than six. In addition, successful transfection of pcDNA3.1-HIF-1α was confirmed by RT–qPCR (Supplementary Fig. 1d). As shown in Fig. 1f, the augmentation of HIF-1α led to an increase in luciferase activity in the WT DACT3-AS1 promoter, which indicated that HIF-1α acted as a transcription activator of DACT3-AS1. Moreover, we conducted ChIP and DNA pull-down assays in HCC cells under normoxia and hypoxia and found that the binding of HIF-1α and the DACT3-AS1 promoter was induced by hypoxia (Fig. 1g–h). Aside from the above investigation, we also utilized CoCl_2_, a chemical inducer of HIF-1α, to stimulate a hypoxic environment. HCC cells were treated with 100 µM CoCl_2_ for 24–48 h, except for the kinetic study. The results of RT–qPCR assays showed that DACT3-AS1 was markedly augmented in HCC cells treated with CoCl_2_, especially in SMMC-7721 and Huh7 cells (Supplementary Fig. 1e). As shown in Supplementary Fig. 1f, the hypoxia mimetic agent CoCl_2_ triggered an increase in DACT3-AS1 expression in a time-dependent manner. With different concentrations of CoCl_2_ solution (0 µM, 100 µM, 200 µM), DACT3-AS1 expression increased accordingly in SMMC-7721 and Huh7 cells (Supplementary Fig. 1g). After 48 h of CoCl_2_ treatment, HIF-1α was found to be significantly elevated in SMMC-7721 and Huh7 cells through RT–qPCR and western blot analyses (Supplementary Fig. 1h). In CoCl_2_-mimicking hypoxia, DACT3-AS1 apparently declined as HIF-1α expression was reduced in SMMC-7721 and Huh7 cells (Supplementary Fig. 1i). The results from ChIP and DNA pull-down assays further confirmed that hypoxia distinctly promoted the interaction between HIF-1α and the DACT3-AS1 promoter in SMMC-7721 and Huh7 cells (Supplementary Fig. 1j–k). In summary, hypoxia-induced HIF-1α acted as the transcriptional activator of DACT3-AS1 in HCC cells.

### DACT3-AS1 is highly expressed in metastatic HCC tissues

**Fig. 2:**
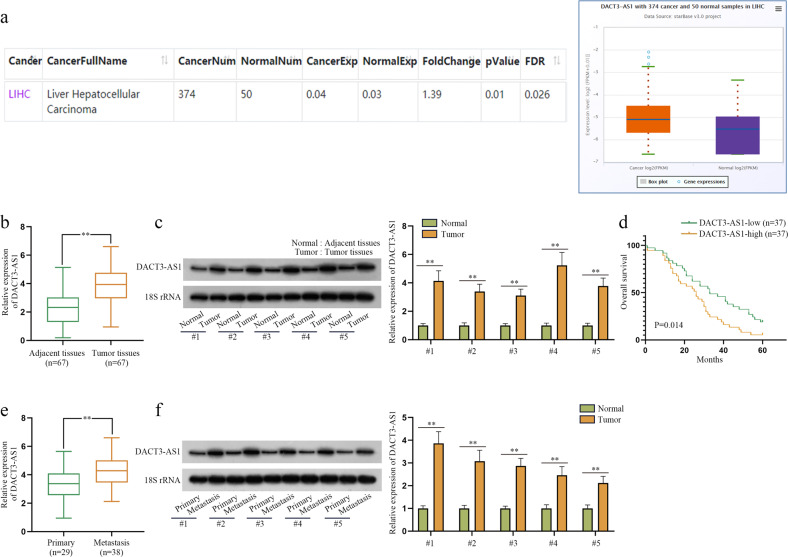
DACT3-AS1 is highly expressed in metastatic HCC tissues. **a** By means of starBase, the expression of DACT3-AS1 in HCC tissues and normal tissues was revealed. **b** RT–qPCR was performed to quantify the expression of DACT3-AS1 in HCC tumor tissues (*n* = 67) and adjacent tissues (*n* = 67). **c** The expression of DACT3-AS1 in HCC tumor tissues and adjacent tissues was examined by northern blot assay. **d** Kaplan–Meier survival analysis was performed to evaluate the effect of DACT3-AS1 on the prognosis of HCC patients. **e** The expression of DACT3-AS1 in primary HCC tumors (*n* = 29) and metastatic HCC tumors (*n* = 38) was quantified by RT–qPCR. **f** Northern blotting was conducted to measure DACT3-AS1 in primary and metastatic HCC tumors. **P < 0.01.

To determine the role of DACT3-AS1 in HCC, we first utilized the starBase database (http://starbase.sysu.edu.cn/) to reveal the expression of DACT3-AS1 in liver hepatocellular carcinoma (LIHC) tissues and normal tissues. The data showed that DACT3-AS1 displayed a remarkably higher level in LIHC tissue than in normal tissues (Fig. 2a). Meanwhile, we collected 67 pairs of HCC tissues and adjacent noncancer tissues. RT–qPCR and northern blotting were performed to reveal the expression of DACT3-AS1 in HCC tissues and noncancer tissues. The results indicated that DACT3-AS1 expression was evidently higher in HCC tissues than in adjacent tissues (Fig. 2b–c). In addition, Kaplan–Meier survival analysis was performed to determine the 5-year overall survival rate of HCC patients with high or low expression of DACT3-AS1. The cutoff applied to divide patients into two groups was the median value of DACT3-AS1 expression in tumor tissues. Patients with DACT3-AS1 expression higher than the median value were divided into the DACT3-AS1-high group, and the rest of them were divided into the DACT3-AS1-low group. Patients with low expression of DACT3-AS1 had a clearly higher survival rate than those with high expression of DACT3-AS1 (Fig. 2d). Among the 67 cases of HCC, tumor metastasis occurred in 38 cases. We also detected the expression of DACT3-AS1 in 38 metastatic HCC tumors and 29 primary HCC tissues. The RT–qPCR and northern blot data demonstrated that DACT3-AS1 presented noticeably higher expression in metastatic tumors than in primary HCC tissues (Fig. 2e–f). Taken together, DACT3-AS1 displayed high expression in HCC tissues, especially in metastatic HCC tumors.

### DACT3-AS1 contributes to HCC cell migration, invasion and EMT in vitro

**Fig. 3:**
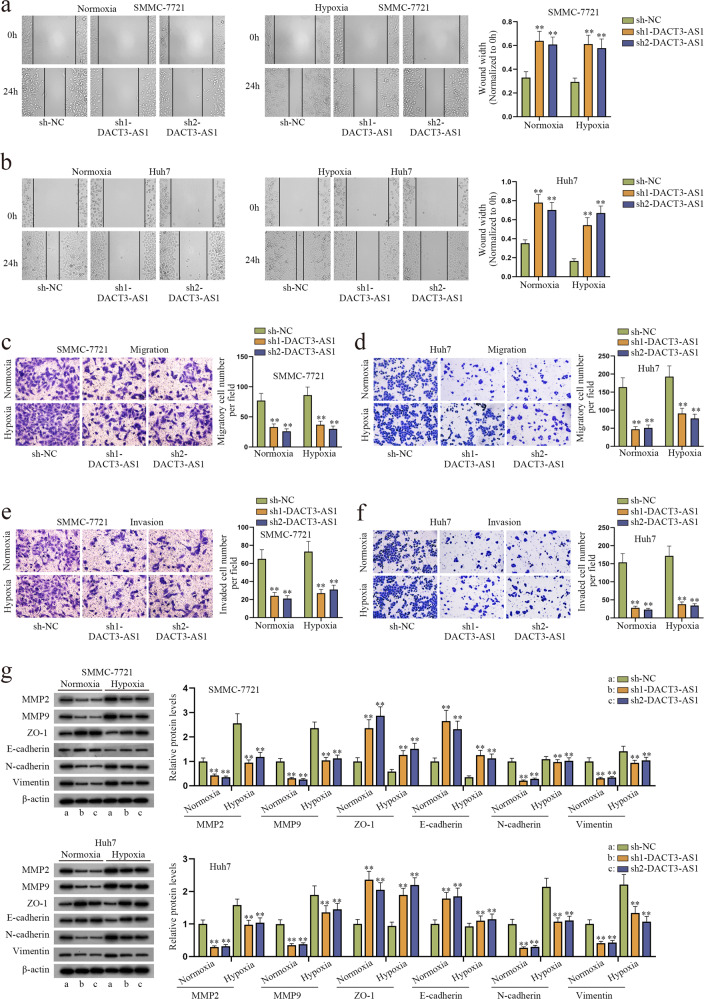
DACT3-AS1 promotes the migration, invasion and EMT of HCC cells. **a**–**d** Wound healing and Transwell migration assays were performed to jointly evaluate the impact of DACT3-AS1 knockdown on the migration of normoxic/hypoxic HCC cells. **e**–**f** Transwell invasion assays were conducted to assess the influence of DACT3-AS1 reduction on the invasion of HCC cells under normoxia/hypoxia. **g** After transfection of DACT3-AS1, the expression of invasion-related and EMT-associated proteins was detected via western blot assays. **P < 0.01.

Based on the clinical data, DACT3-AS1 was conspicuously highly expressed in HCC tissues in comparison to adjacent nontumor tissues. Moreover, metastatic HCC tumors harbored an evidently higher expression of DACT3-AS1 than HCC primary tumors, which indicated that DACT3-AS1 might exert an influential role on HCC metastasis. Hence, we probed the effect of DACT3-AS1 on cell migratory and invasive abilities as well as the EMT process in HCC. First, HCC cells were cultured for 24–48 h under hypoxic conditions with 5% CO_2_, 1% O_2_ and 94% N_2_. Next, we knocked down DACT3-AS1 and chose sh1-DACT3-AS1 and sh2-DACT3-AS1 for the subsequent assays due to their higher knockdown efficacy (Supplementary Fig. 2a). Subsequently, a series of functional assays were carried out in HCC cells under normoxia or hypoxia. As shown in wound healing assays, the migratory ability of normoxic and hypoxic HCC cells was weakened when DACT3-AS1 was downregulated (Fig. 3a–b). The results of Transwell assays demonstrated that the number of migrated and invasive cells was dramatically decreased after DACT3-AS1 knockdown under normoxia and hypoxia (Fig. 3c–f). Furthermore, western blot assays were conducted to detect the expression of invasion (MMP2 and MMP9) and EMT markers (ZO-1, E-cadherin, N-cadherin and Vimentin). Cell invasion and EMT were impeded in HCC cells transfected with sh-DACT3-AS1 (Fig. 3g). The same functional assays in CoCl_2_-induced hypoxic HCC cells were also carried out. PBS treated cells served as the control group. The outcomes were consistent with those in HCC cells cultured in a hypoxic environment (Supplementary Fig. 2b–h). In summary, DACT3-AS1 could accelerate the process of migration, invasion and EMT in HCC cells.

### DACT3-AS1 facilitates metastasis of HCC

**Fig. 4:**
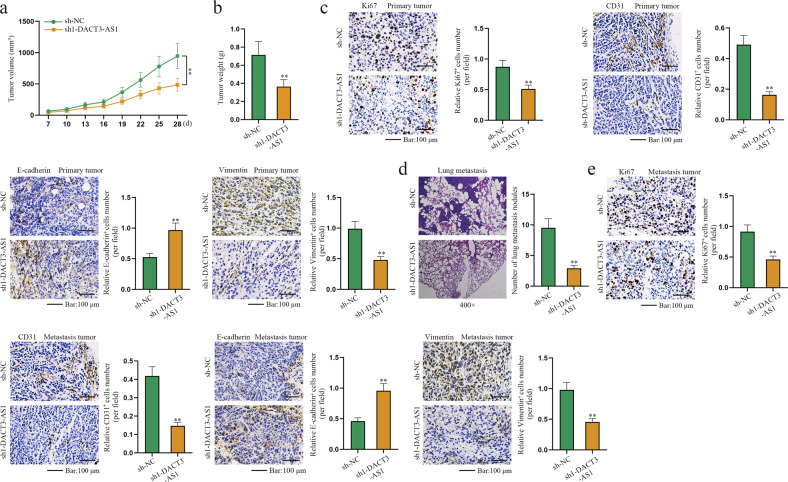
DACT3-AS1 promotes metastasis in HCC. **a**–**b** The volume and weight of xenograft tumors were measured. **c** IHC assays were carried out to detect the expression of proliferation, neoangiogenesis and EMT markers in sh-NC and sh1-DACT3-AS1 groups of primary tumors. **d** The number of lung metastasis nodules was counted in the sh-NC and sh1-DACT3-AS1 groups. **e** IHC assays were performed to detect the expression of Ki76, CD31, E-cadherin and Vimentin in sh-NC and sh1-DACT3-AS1 groups of metastatic tumors. **P < 0.01.

In vivo assays were designed and carried out. The tumor volume of mice was monitored every 3 days after the first measurement on the 7th day of cell injection. The collected data are displayed as a line chart, which indicated that the tumors in the sh1-DACT3-AS1 group were smaller than those in the control group (Fig. 4a). After the tumors were excised, tumor weight was measured. As shown in Fig. 4b, DACT3-AS1 knockdown dramatically reduced the tumor weight. Meanwhile, IHC assays were conducted to analyze the expression of Ki-67, CD31, E-cadherin and Vimentin in primary HCC tissues. The outcomes revealed that the expression of Ki-67, CD31 and Vimentin declined, while that of E-cadherin rose due to DACT3-AS1 reduction (Fig. 4c). Afterward, xenografts from SMMC-7721 injections were isolated and implanted into the liver to construct orthotopic xenografts. The quantity of lung metastatic lesions in the sh1-DACT3-AS1 group was much smaller than that in the sh-NC group (Fig. 4d). Moreover, the expression of Ki-67, CD31 and Vimentin was confirmed to be reduced, while that of E-cadherin was increased after DACT3-AS1 downregulation (Fig. 4e). In summary, DACT3-AS1 could promote HCC tumor metastasis.

### DACT3-AS1 downregulates FOXA3 to strengthen HCC cell migratory, invasive and EMT capabilities

**Fig. 5:**
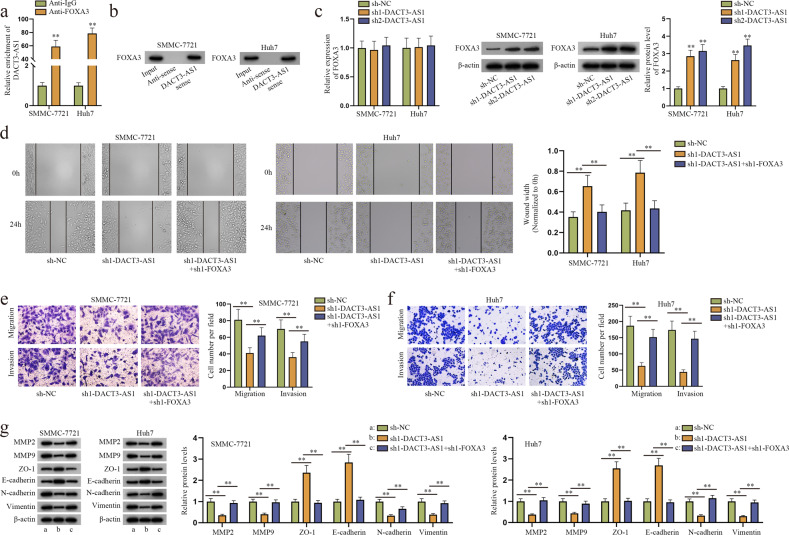
DACT3-AS1 interacts with FOXA3 to regulate HCC cell migration, invasion and EMT. **a** After RIP assays, RT–qPCR analyses were conducted to quantify the enrichment of DACT3-AS1. **b** After RNA pull-down assays, western blot assays were performed to detect the protein level of FOXA3 pulled down by DACT3-AS1 sense. **c** The mRNA and protein levels of FOXA3 were determined by RT–qPCR and western blot assays after DACT3-AS1 was knocked down. **d** Wound healing assays were carried out to evaluate the migration of HCC cells transfected with different plasmids (sh-NC, sh1-DACT3-AS1, sh1-DACT3-AS1 + sh1-FOXA3). **e**–**f** Through transwell assays, the migratory and invasive abilities of HCC cells receiving different treatments were assessed. **g** Western blot assays were performed to measure the expression of invasion-related and EMT-associated proteins under different conditions. **P < 0.01.

In this section, the functional mechanism of DACT3-AS1 in HCC cells was investigated. LncRNAs have been revealed to participate in the cis modulation of target genes located at or near the same genomic locus^30,31^. As a result, we speculated that DACT3-AS1 might also regulate the expression of its target mRNA DACT3. However, the RT–qPCR results suggested that DACT3 expression was hardly changed in HCC cells with DACT3-AS1 depletion (Supplementary Fig. 3a), indicating that DACT3-AS1 did not exert its function by modulating the neighboring mRNA DACT3. Moreover, we found that DACT3-AS1 was situated at 19q13.32 on the chromosome and near some protein-coding genes, such as BHMG1, FBXO46, GPR4 and FOXA3. Among those genes, FOXA3 was predicted to interact with DACT3-AS1 by means of bioinformatics tools (Supplementary Fig. 3b). In addition, the expression of FOXA3 in hypoxic or CoCl_2_-treated cells was much lower than that in their control groups (Supplementary Fig. 3c). Therefore, the relationship of DACT3-AS1 and FOXA3 in HCC cells attracted our attention and was further explored. The results of RIP and RNA pull-down assays revealed that there was a binding interaction between DACT3-AS1 and FOXA3 in HCC cells (Fig. 5a–b). The following RT–qPCR and western blot analysis showed that DACT3-AS1 knockdown could increase the expression of FOXA3 protein while having no impact on that of FOXA3 mRNA in HCC cells (Fig. 5c). After confirming the successful transfection of sh1/2/3-FOXA3 and selecting the most efficient sh1-FOXA3 (Supplementary Fig. 3d), we performed functional rescue assays. As revealed in wound healing (Fig. 5d), Transwell (Fig. 5e–f) and western blot assays (Fig. 5g), HCC cell migration, invasion and EMT processes were all hindered due to DACT3-AS1 decline, while the results were the opposite after FOXA3 reduction. In brief, DACT3-AS1 diminished FOXA3 expression to facilitate migration and invasion along with EMT of HCC cells.

### HDAC2 could reduce FOXA3 expression by inhibiting its acetylation level in HCC cells

**Fig. 6:**
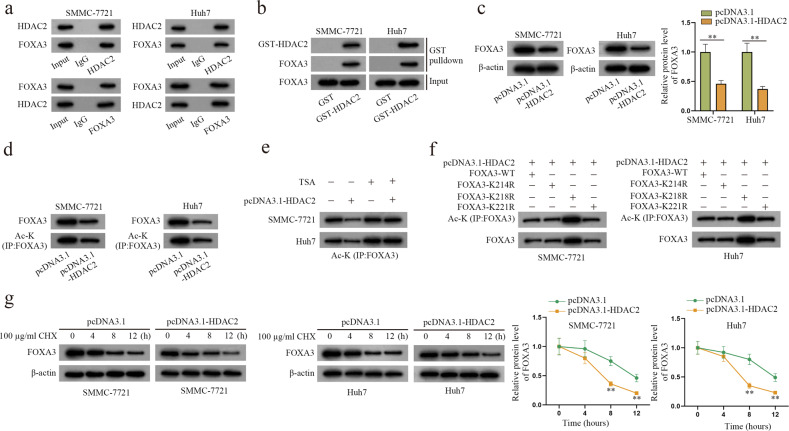
HDAC2 regulates FOXA3 acetylation. **a**–**b** Co-IP and GST pull-down assays were implemented to analyze the interaction between HDAC2 and FOXA3 in HCC cells. **c** Western blot assays were performed to detect the protein level of FOXA3 in HCC cells transfected with pcDNA3.1-HDAC2. **d** IP-WB was performed to confirm the effect of HDAC2 overexpression on the acetylation of FOXA3. **e** IP-WB was conducted to determine the influence of HDAC2 augmentation on FOXA3 acetylation in HCC cells treated with TSA. **f** Through IP-WB assays, the changes in FOXA3 acetylation were examined as the predicted binding sites were mutated. **g** The protein level of FOXA3 was detected in HCC cells treated with 100 µg/ml CHX. **P < 0.01.

We next delved into the specific mechanism by which DACT3-AS1 regulates FOXA3. RNA-protein interactions have a vital effect on diverse biological processes^32^. Hence, we presumed that DACT3-AS1 might also interact with certain proteins to modulate FOXA3. With the help of the HitPredict (http://www.hitpredict.org/) and RPIseq (http://pridb.gdcb.iastate.edu/RPISeq/) websites, HDAC2 was identified as the common binding protein of DACT3-AS1 and FOXA3 (Supplementary Fig. 4a–b). UALCAN (http://ualcan.path.uab.edu) was utilized, and the search results revealed that HDAC2 displayed higher expression in LIHC tissues than in normal tissues (Supplementary Fig. 4c). RT–qPCR and western blot assays were then implemented to detect the expression of HDAC2 in HCC cells under normoxia or hypoxia. HDAC2 expression was apparently elevated in hypoxic cells (Supplementary Fig. 4d). Therefore, HDAC2 became the subject of the following experiments. In Co-IP and GST pull-down assays, HDAC2 was verified to interact with FOXA3 in HCC cells (Fig. 6a–b). Prior to western blot, the transfection of pcDNA3.1-HDAC2 was validated to be effective (Supplementary Fig. 4e). Then, the results showed that HDAC2 augmentation led to diminished FOXA3 in HCC cells (Fig. 6c). According to the current literature, HDAC2 is a type of deacetylase^33^. Moreover, the PhosphositePlus (www.phosphosite.org) database was utilized, and FOXA3 had acetylation sites (K214, K218 and K221) (Supplementary Fig. 4f). Consequently, the effect of HDAC2 on the FOXA3 acetylation level was investigated. The outcomes of IP-WB revealed that HDAC2 elevation reduced the level of acetylated lysine (Ac-K) on FOXA3 in HCC cells (Fig. 6d). In addition, the decreased level of Ac-K was restored by treatment with 2 µm trichostatin A (TSA), an inhibitor of histone deacetylase, in IP-WB, signifying that HDAC2 could promote FOXA3 deacetylation in HCC cells (Fig. 6e). To determine the functional sites of HDAC2 on FOXA3 acetylation, the predicted acetylation sites on FOXA3 were mutated before IP-WB assay. The data showed that HDAC2 overexpression led to an increase in FOXA3 acetylation in HCC cells only when K218 sites were mutated (Fig. 6f), indicating that K218 was the effective site. After HCC cells were treated with CHX, western blot assays were performed to detect the expression of FOXA3 at different time points (Fig. 6g). The results suggested that the HDAC2 increase accelerated FOXA3 protein degradation. In summary, HDAC2 increased FOXA3 deacetylation levels to reduce FOXA3 expression in HCC cells.

### DACT3-AS1 could enhance the binding between HDAC2 and FOXA3 in HCC cells

**Fig. 7:**
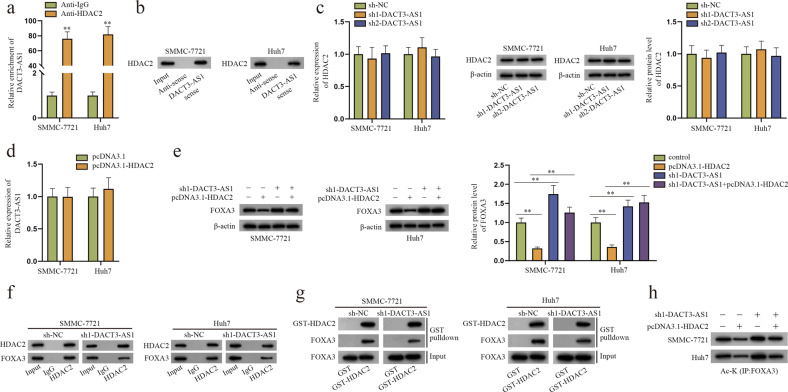
DACT3-AS1 promotes the binding between HDAC2 and FOXA3. **a** After RIP assays, RT–qPCR was performed to quantify the enrichment of DACT3-AS1. **b** RNA pull-down and western blot assays were carried out to confirm the binding between HDAC2 and DACT3-AS1 sense. **c** RT–qPCR and western blotting were performed to measure the expression of HDAC2 after DACT3-AS1 knockdown in HCC cells. **d** RT–qPCR was performed to detect the expression of DACT3-AS1 after HDAC2 was overexpressed in HCC cells. **e** Western blot assays were performed to examine the protein level of FOXA3 in HCC cells transfected with different plasmids. **f** Co-IP experiments were performed to assess the interaction between HDAC2 and FOXA3 in HCC cells. **g** GST pull-down and western blot assays were employed to determine the impact of DACT3-AS1 downregulation on the binding of FOXA3 and HDAC2. **h** IP-WB assay was utilized to analyze the acetylation level of FOXA3 in HCC cells with different treatments. **P < 0.01.

To determine the role of DACT3-AS1 in the affinity of HDAC2 and FOXA3, RIP and RNA pull-down assays were conducted to reveal the binding relationship between DACT3-AS1 and HDAC2. The results showed that DACT3-AS1 could bind to HDAC2 (Fig. 7a–b). In addition, the results of RT–qPCR and western blot assays demonstrated that DACT3-AS1 knockdown had no effect on the expression of HDAC2 (Fig. 7c). Meanwhile, HDAC2 upregulation was discovered to have no effect on the expression of DACT3-AS1 through RT–qPCR in HCC cells (Fig. 7d). Considering these data, we verified that DACT3-AS1 simply interacted with HDAC2, but they did not affect the expression of each other. As depicted in the following western blot assay, transfection of pcDNA3.1-HDAC2 decreased the expression of FOXA3, while cotransfection of sh1-DACT3-AS1 offset the effect in HCC cells (Fig. 7e). Subsequent Co-IP and GST pull-down assays jointly proved that DACT3-AS1 reduction weakened binding between HDAC2 and FOXA3 in HCC cells (Fig. 7f–g). Moreover, the IP-WB data proved that the acetylation level of FOXA3 was reduced due to HDAC2 augmentation but was restored by cotransfection of sh1-DACT3-AS1 (Fig. 7h). In conclusion, DACT3-AS1, HDAC2 and FOXA3 form a complex to inhibit FOXA3 expression.

### FOXA3 serves as a transcriptional inhibitor of PKM2

**Fig. 8:**
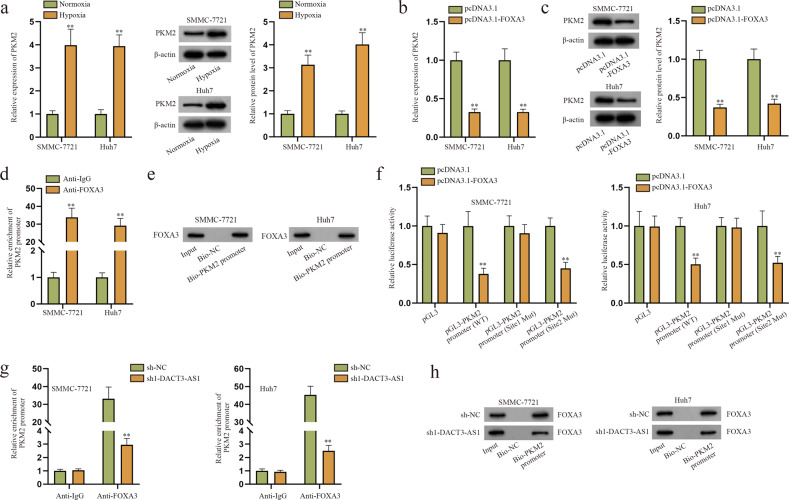
FOXA3 inhibits PKM2 expression at the transcriptional level. **a** RT–qPCR and western blot assays were performed to examine the expression of PKM2 in normoxic and hypoxic cells. **b**–**c** After FOXA3 was overexpressed, the expression of PKM2 was detected via RT–qPCR and western blot. **d**–**e** ChIP and DNA pull-down assays were implemented to unravel the affinity between FOXA3 and the PKM2 promoter. **f** The binding sites of FOXA3 and PKM2 were verified in a luciferase reporter assay. **g**–**h** ChIP and DNA pull-down assays were conducted in HCC cells transfected with sh1-DACT3-AS1 to evaluate the change in the interaction between FOXA3 and PKM2. **P < 0.01.

According to previous studies, PKM2 transcription is activated by HIF-1α under hypoxia^34,35^. PKM2 has also been identified to participate in HCC tumor growth and metastasis^36^, for which we hypothesized that PKM2 might engage in hypoxia-induced HCC metastasis. Using the JASPAR database, we predicted the potential binding site between FOXA3 and the PKM2 promoter (Supplementary Fig. 5a). Hence, we tried to determine the regulatory relationship between FOXA3 and PKM2. Predicted Site 1 and Site 2 were investigated in the following experiments, as their confidence levels were high (scores > 8). Through RT–qPCR and western blot assays, PKM2 was significantly upregulated in hypoxic HCC cells (Fig. 8a). After testing the overexpression efficiency of pcDNA3.1-FOXA3 (Supplementary Fig. 5b), we further detected the RNA and protein levels of PKM2 in response to FOXA3 overexpression and found that FOXA3 augmentation reduced the expression of PKM2 in HCC cells (Fig. 8b–c). ChIP and DNA pull-down assays were performed to jointly certify the binding affinity of the PKM2 promoter and FOXA3 (Fig. 8d–e). The results of subsequent luciferase reporter assays not only indicated that the PKM2 promoter could interact with FOXA3 at predicted Site 1 but also revealed that FOXA3 acted as a transcriptional inhibitor of PKM2 in HCC cells (Fig. 8f). In addition, we further confirmed that the downregulation of DACT3-AS1 weakened the binding of the PKM2 promoter and FOXA3 (Fig. 8g–h). In summary, FOXA3, as a transcription factor, could repress the transcription of PKM2 in HCC cells.

### FOXA3 hinders HCC cell migration, invasion and EMT by downregulating PKM2

**Fig. 9:**
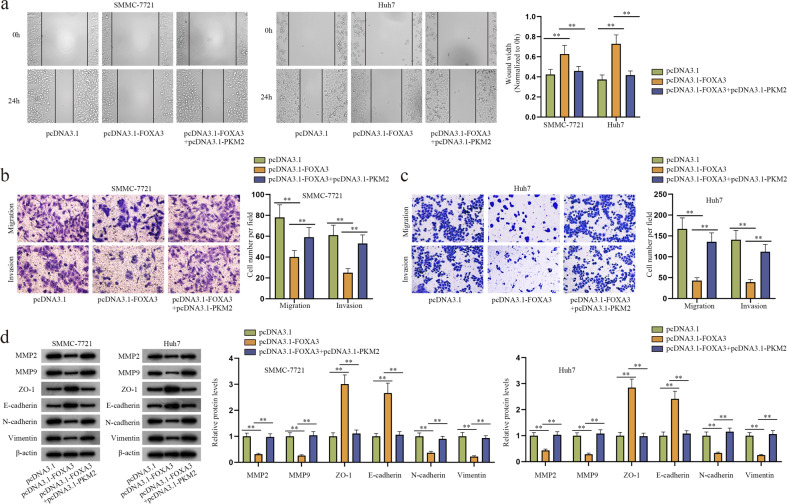
FOXA3 impedes HCC migration, invasion and EMT by diminishing PKM2. Functional assays were performed in HCC cells transfected with pcDNA3.1, pcDNA3.1-FOXA3 or pcDNA3.1-FOXA3 + pcDNA3.1-PKM2. **a** A wound healing assay was performed to detect cell migratory ability in HCC cells with different transfections. **b**–**c** Transwell assays were conducted to measure cell migratory and invasive capabilities under different conditions. **d** The expression of invasion- and EMT-related proteins was detected by western blot assays. **P < 0.01.

To elucidate the effects of FOXA3 and PKM2 on cell processes, functional rescue experiments were conducted. HCC cells were divided into three groups and transfected with pcDNA3.1, pcDNA3.1-FOXA3, or pcDNA3.1-FOXA3 + pcDNA3.1-PKM2. The successful transfection of pcDNA3.1-PKM2 was determined by RT–qPCR (Supplementary Fig. 5c). By wound healing and Transwell assays, we confirmed that the suppressed migratory and invasive abilities of HCC cells resulting from FOXA3 overexpression were restored by simultaneous upregulation of PKM2 (Fig. 9a–c). The western blot results also suggested that the expression of key proteins related to invasion and EMT declined after transfection of pcDNA3.1-FOXA3, while this decrease was further recovered by the PKM2 increase (Fig. 9d). In short, the suppressive effects of FOXA3 overexpression on HCC cell migration, invasion and EMT could be counteracted by PKM2 upregulation.

### Coexpression analysis of the RNA-RNA interactions in LIHC tissues

In this section, we mainly utilized the starBase database to analyze the expression correlation between different target genes in LIHC tissues. First, HIF-1α and DACT3-AS1 were found to have a positive correlation with each other in LIHC tissues (Supplementary Fig. 6a). Moreover, it was also predicted that the expression of HIF-1α was positively correlated with that of HDAC2 in LIHC tissues (Supplementary Fig. 6b). Subsequently, the correlation of HIF-1α and FOXA3 in LIHC tissues was also analyzed. HIF-1α and FOXA3 expression had a negative relationship in LIHC tissues (Supplementary Fig. 6c). Furthermore, coexpression analysis for HIF-1α and PKM2 in LIHC tissues was conducted, and we detected a positive correlation (Supplementary Fig. 6d). Next, the positive relationship between DACT3-AS1 and HDAC2/PKM2 in LIHC tissues was also revealed on the starBase website (Supplementary Fig. 6e–f). In addition, we analyzed the coexpression of PKM2 and HDAC2/FOXA3. The search results showed that PKM2 had a positive correlation with HDAC2 but had a negative correlation with FOXA3 in LIHC tissues (Supplementary Fig. 6g–h).

## Discussion

HCC is a common malignancy with high mortality worldwide^37^. Approximately 70–80% of patients are diagnosed at an advanced stage and can receive palliative treatments^38^. Based on recent studies, hypoxia and its molecular responses have been recognized to correlate with poor prognosis and increased mortality^39,40^. Studies have investigated the impacts and relevant mechanism of the hypoxic microenvironment and HIFs in HCC^41,42^. Moreover, the impact of lncRNAs on HCC under hypoxia has been elucidated. For instance, hypoxia-induced lncRNA-NEAT1 contributes to the growth of HCC by modulating miR-199a-3p/UCK2^28^. NR2F1-AS1 knockdown restricts hypoxia-induced migration of HCC cells via the regulation of miR-140 and HK2^43^. In this study, we conducted RT–qPCR to determine the research target. DACT3-AS1 was selected for its significantly higher expression in hypoxic HCC cells than in normoxic cells. Subsequently, this study revealed that upregulation of DACT3-AS1 was induced by hypoxia in HCC cells in a time-/dose-dependent manner.

Extensive studies illustrate that hypoxia-inducible factors (HIFs) represent key proteins that affect tumor responses to hypoxia. Specifically, HIF-1α functions as a regulator of hypoxia-induced transcription to promote tumor glycolysis, angiogenesis, invasion and metastasis^44^. Studies have investigated the impacts of the interaction between HIF-1α and lncRNAs on HCC under hypoxia^42^. HIF1A facilitates the transcription of lncRNA RAET1K to affect hypoxia-induced glycolysis in HCC cells via miR-100-5p^42^. HIF-1α upregulates the expression of lncRNA NEAT1 as a transcription activator to promote the development of HCC under hypoxia^45^. In this research, we performed RT–qPCR and western blot assays and found that HIF-1α was significantly highly expressed in hypoxic HCC cells and positively regulated DACT3-AS1 expression under hypoxia. Mechanistic analyses verified that HIF-1α could interact with the DACT3-AS1 promoter at predicted Site 1, transcriptionally activating DACT3-AS1 in hypoxic HCC cells. In addition, CoCl_2_ was utilized to simulate the hypoxic environment, and in CoCl_2_-induced hypoxic HCC cells, the same conclusions were drawn. Subsequently, the functional role of DACT3-AS1 in HCC cells under normoxia and hypoxia was probed. The collected wound healing, Transwell and western blot data jointly reflected that DACT3-AS1 played an oncogenic role in HCC cells, especially under hypoxia. As DACT3-AS1 expression declined, hypoxic HCC cell migration, invasion and EMT were impeded accordingly.

In recent years, many lncRNAs have been revealed to function as positive or negative modulators of nearby genes^46^. Aberrantly expressed lncRNAs and their associated neighboring coding genes play a crucial role in the development of cancer^47^. LncRNA WWOX-AS1 competitively binds to miR-20b-5p in HCC and impedes its progression by increasing the expression of WWOX^48^. GPC3-AS1 positively regulates its nearby gene GPC3 to strengthen the proliferative and migratory abilities of cervical cancer cells^49^. Furthermore, DACT3-AS1 was also discovered to interact with one of its nearby genes, FOXA3, by means of bioinformatics tools and related experiments. In addition, the functional assay data revealed that the suppressive effects of sh-DACT3-AS1 on cell migration, invasion and EMT could be counteracted by FOXA3 knockdown. Previous studies have revealed that lncRNAs can modulate their nearby protein-coding genes through interactions with proteins^50^. In this study, HDAC2 was identified as the shared interacting protein of DACT3-AS1 and FOXA3. HDAC2 has been reported to restrain FBP1 expression and facilitate HCC cell proliferation^51^, whereas the relationship of FOXA3 and HDAC2 has not been revealed thus far. Herein, the mechanistic analyses illustrated that FOXA3 could bind to HDAC2. This binding could lead to a decrease in the FOXA3 acetylation level, which further reduced FOXA3 expression. Subsequently, DACT3-AS1 was proven to interact with HDAC2, and DACT3-AS1 could enhance the affinity between FOXA3 and HDAC2. The above data together showed that DACT3-AS1 recruited HDAC2 to decrease the expression of FOXA3.

As a vital modulator of the growth and metastasis of cancer cells, PKM2 has been proven to be highly expressed in diverse human cancers, including HCC^52^. FOXA3 is a transcription factor^53^. In the present study, FOXA3 was proven to bind to the PKM2 promoter and reduce the level of PKM2 as a transcription activator, which has not been uncovered in other studies. The following functional rescue experiments indicated that FOXA3 could restrict cell migration, invasion and EMT by diminishing PKM2 expression. As previous studies have suggested, lncRNA-SOX2OT contributes to HCC invasion and metastasis by heightening the expression of PKM2^54^. In line with the former finding, this research also revealed that PKM2 acted as an oncogene in HCC cells.

In summary, our study first unveiled that DACT3-AS1 was transcriptionally activated by HIF-1α in HCC cells under hypoxia. In terms of the regulatory mechanism, DACT3-AS1 enhanced the binding ability between FOXA3 and HDAC2 to decrease the expression of FOXA3. Additionally, FOXA3 reduced PKM2 expression at the transcriptional level. Based on the gathered clinical data, DACT3-AS1 manifested high expression in metastatic HCC tumors, and HCC patients with low DACT3-AS1 expression had a more favorable prognosis than those with high DACT3-AS1 expression. An in vivo study further revealed that DACT3-AS1 promoted HCC tumor metastasis. Although other downstream molecular mechanisms of DACT3-AS1 were not discussed in this study, we still hope this study will increase the understanding of HCC and provide valid evidence to develop potential therapeutic targets for HCC treatment.

## Supplementary information

Supplementary information
