# Supplementary material for: Hypoxia-induced LncRNA DACT3-AS1 upregulates PKM2 to promote metastasis in hepatocellular carcinoma through the HDAC2/FOXA3 pathway
Source: Exp Mol Med. 2022 Jun 28;54(6):848–60. doi: 10.1038/s12276-022-00767-3 (PMC9256752; doi:10.1038/s12276-022-00767-3)

Supplementary Fig. 1

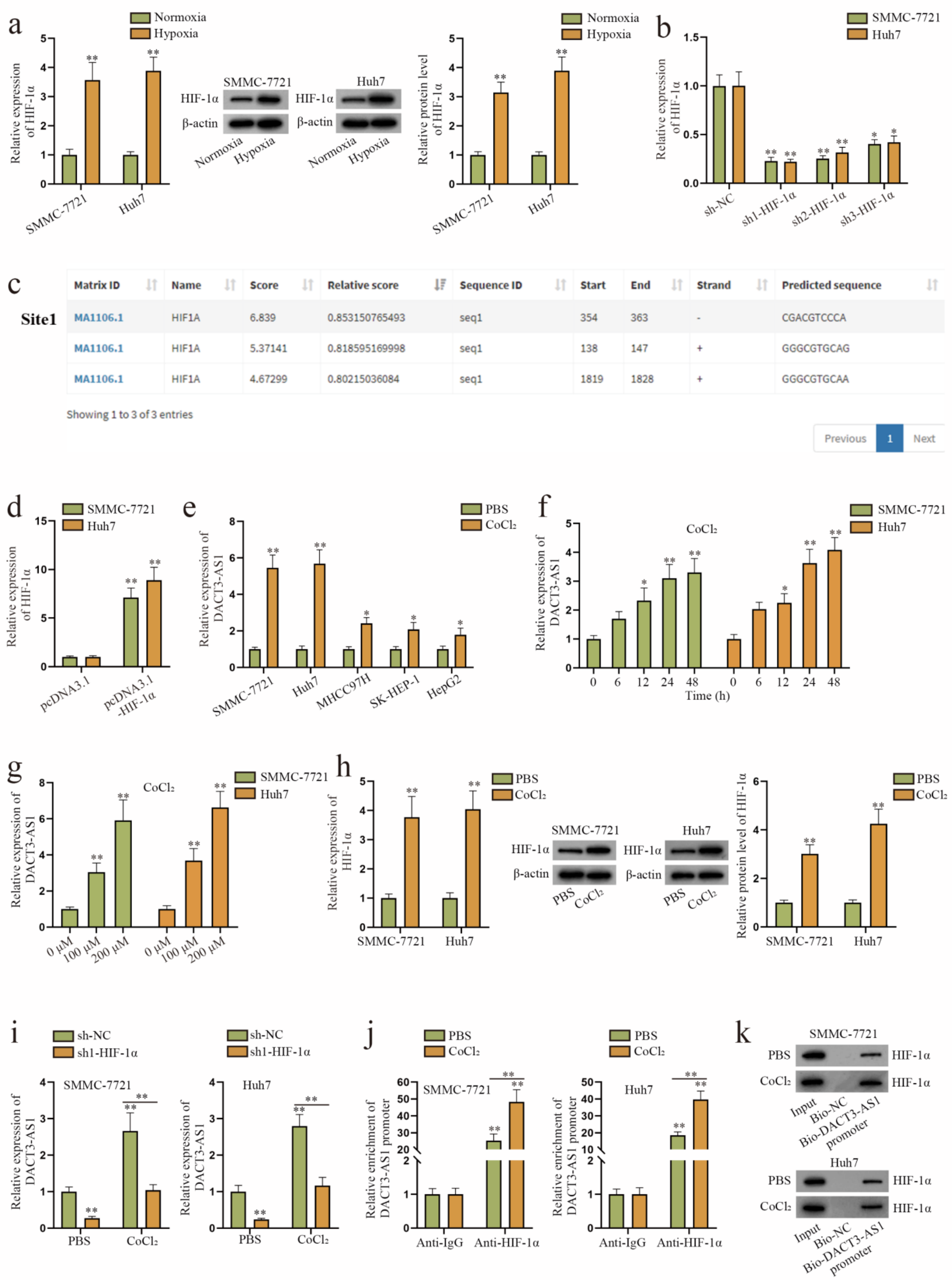

(a) The expression of HIF-1α was analyzed by means of RT-qPCR and western blot in HCC cells under

normoxia/hypoxia. (b) Knockdown efficiency of sh1/2/3-HIF-1α was evaluated in RT-qPCR. (c) The binding sites of HIF-1α and DACT3-AS1 promoter were predicted from JASPAR website. (d) Overexpression efficiency of pcDNA3.1-HIF-1α was confirmed via RT-qPCR. (e) RT-qPCR was employed to quantify the expression of DACT3-AS1 in HCC cells treated with PBS/$CoCl_2$. (f) RT-qPCR was performed to examine the expression of DACT3-AS1 in HCC cells treated with $CoCl_2$ for 6/12/24/48 hours. (g) After HCC cells were cultured with $CoCl_2$ in different concentration, RT-qPCR was implemented to detect the expression of DACT3-AS1. (h) RT-qPCR and western blot assays were applied to ascertain the expression of HIF-1α in HCC cells with treatment of PBS or $CoCl_2$. (i) Expression of DACT3-AS1 was tested in PBS/$CoCl_2$-treated cells after HIF-1α reduction. (j-k) ChIP and DNA pull-down assays were carried out to uncover the binding relation between DACT3-AS1 and HIF-1α under different conditions. *$P < 0.05$, **$P < 0.01$.

Supplementary Fig.2

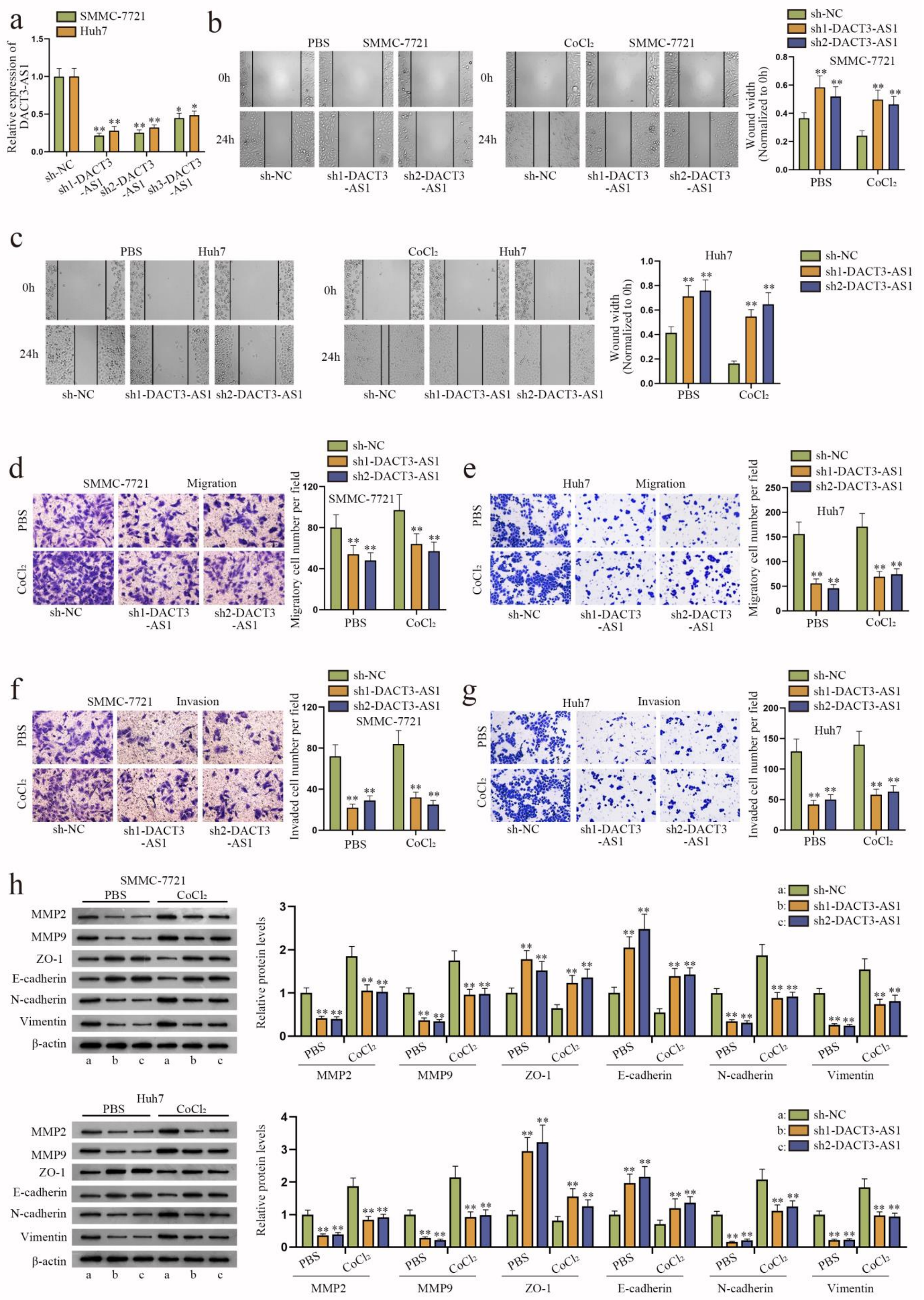

(a) The knockdown efficiency of sh1/2/3-DACT3-AS1 was examined by RT-qPCR. (b-e) After

DACT3-AS1 down-regulation, wound healing and transwell migration assays were operated to evaluate migration of HCC cells under $CoCl_2$-induced hypoxic environment. (f-g) Transwell invasion assays were conducted to assess invasive ability of $CoCl_2$-induced hypoxic HCC cells transfected with sh1/2-DACT3-AS1. (h) Western blot assays were done to measure the protein level of invasion and EMT markers after transfection of sh1/2-DACT3-AS1 in HCC cells under $CoCl_2$-mimicking hypoxia. *$P < 0.05$, **$P < 0.01$.

Supplementary Fig.3

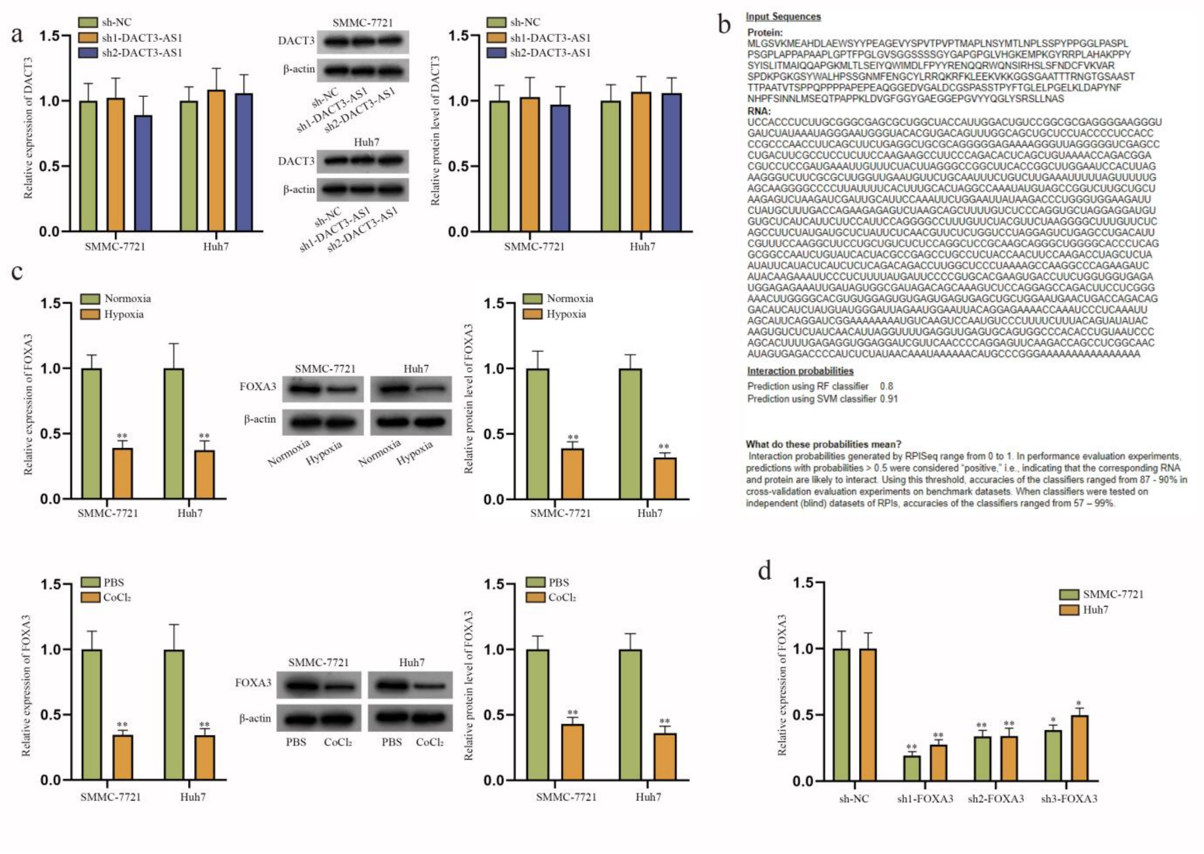

(a) RT-qPCR and western blot assays were performed to examine the mRNA and protein levels of DACT3 in HCC cells transfected with sh1/2-DACT3-AS1. (b) RPISeq website was applied to analyze the possibility of DACT3-AS1-FOXA3 interaction. (c) The RNA and protein levels of FOXA3 were detected in normoxic/hypoxic HCC cells. (d) The knockdown efficiency of sh1/2/3-FOXA3 in HCC cells were tested by RT-qPCR. *P < 0.05, **P < 0.01.

Supplementary Fig.4

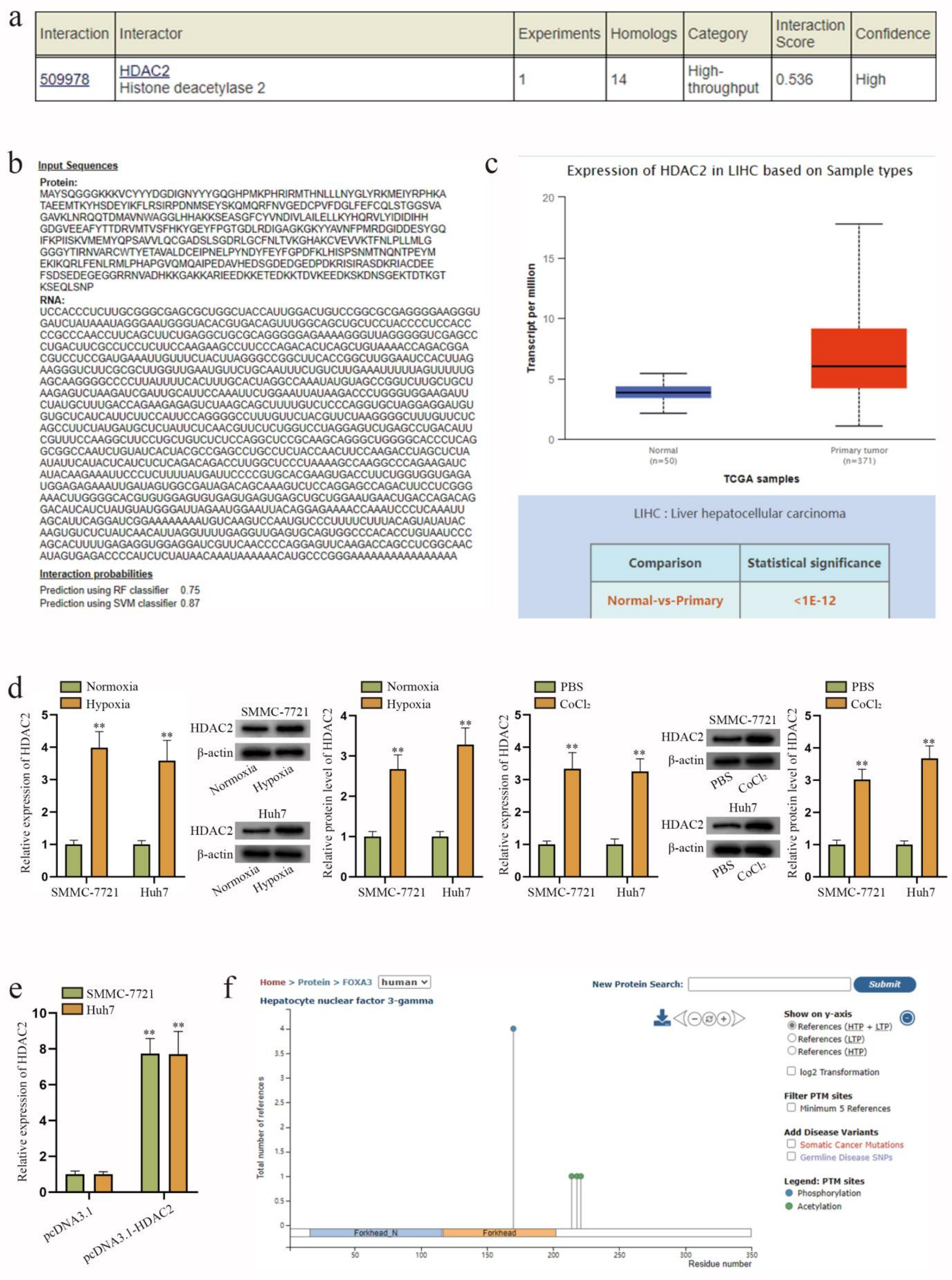

(a) Hitpredict website was used to analyze the protein likely interacting with FOXA3. (b) RPISeq website

was utilized to predict the possibility that DACT3-AS1 might interact with HDAC2. (c) UALCAN database was employed to find the expression of HDAC2 in normal and LIHC tumor tissues. (d) RT-qPCR and western blot assays were conducted to detect the expression of HDAC2 in $CoCl_2$-induced hypoxic HCC cells. (e) The overexpression efficiency of pcDNA3.1-HDAC2 was examined by RT-qPCR. (f) With the help of PhosphositePlus website, the acetylation sites on FOXA3 were predicted. **$P < 0.01$.

Supplementary Fig.5

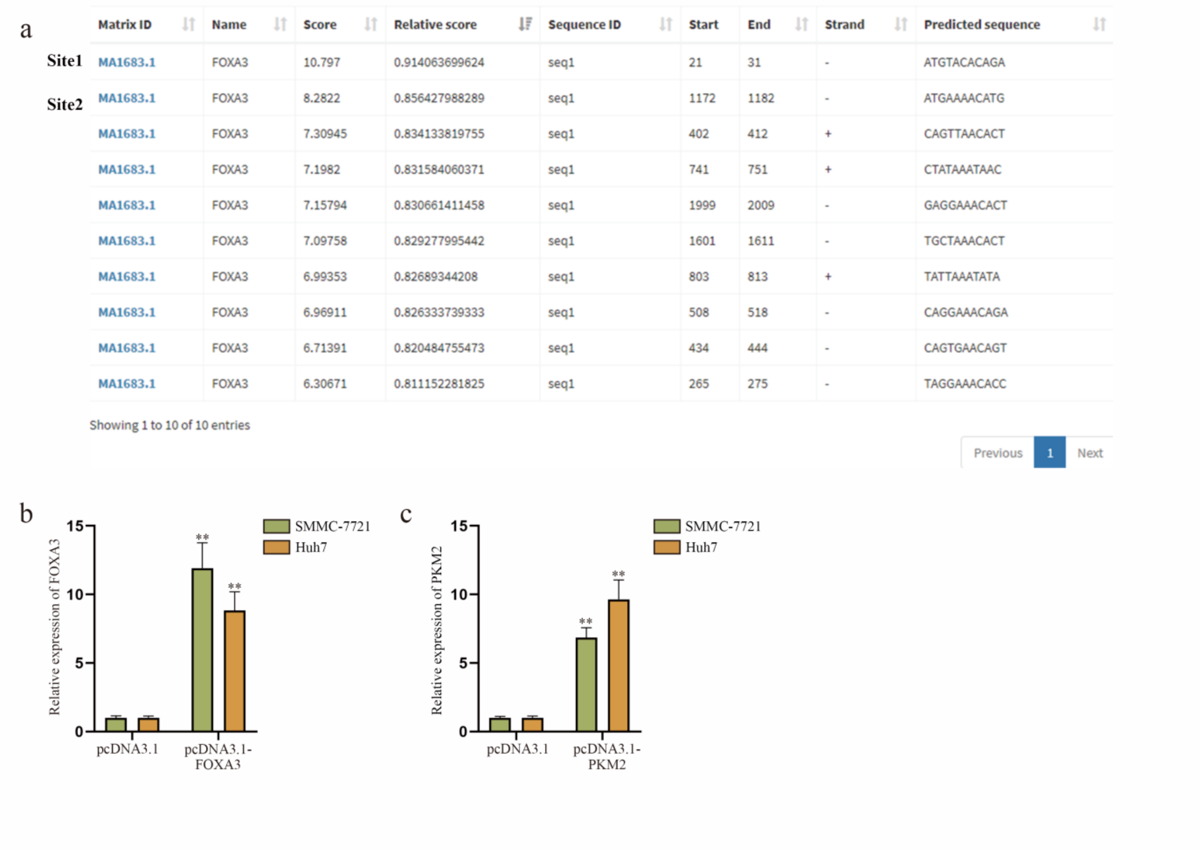

a

	Matrix ID	Name	Score	Relative score	Sequence ID	Start	End	Strand	Predicted sequence
Site1	MA1683.1	FOXA3	10.797	0.914063699624	seq1	21	31	-	ATGTACACAGA
Site2	MA1683.1	FOXA3	8.2822	0.856427988289	seq1	1172	1182	-	ATGAAAACATG
	MA1683.1	FOXA3	7.30945	0.834133819755	seq1	402	412	+	CAGTTAACACT
	MA1683.1	FOXA3	7.1982	0.831584060371	seq1	741	751	+	CTATAAATAAC
	MA1683.1	FOXA3	7.15794	0.830661411458	seq1	1999	2009	-	GAGGAAACACT
	MA1683.1	FOXA3	7.09758	0.829277995442	seq1	1601	1611	-	TGCTAAACACT
	MA1683.1	FOXA3	6.99353	0.82689344208	seq1	803	813	+	TATTAAATATA
	MA1683.1	FOXA3	6.96911	0.826333739333	seq1	508	518	-	CAGGAAACAGA
	MA1683.1	FOXA3	6.71391	0.820484755473	seq1	434	444	-	CAGTGAACAGT
	MA1683.1	FOXA3	6.30671	0.811152281825	seq1	265	275	-	TAGGAAACACC

Showing 1 to 10 of 10 entries

Previous 1 Next

(a) JASPAR was utilized to project the binding sites between FOXA3 and PKM2. (b) Transfection efficiency of pcDNA3.1-FOXA3 was detected by means of RT-qPCR. (c) RT-qPCR was employed to assess the overexpression effectiveness of pcDNA3.1-PKM2. **P < 0.01.

Supplementary Fig.6

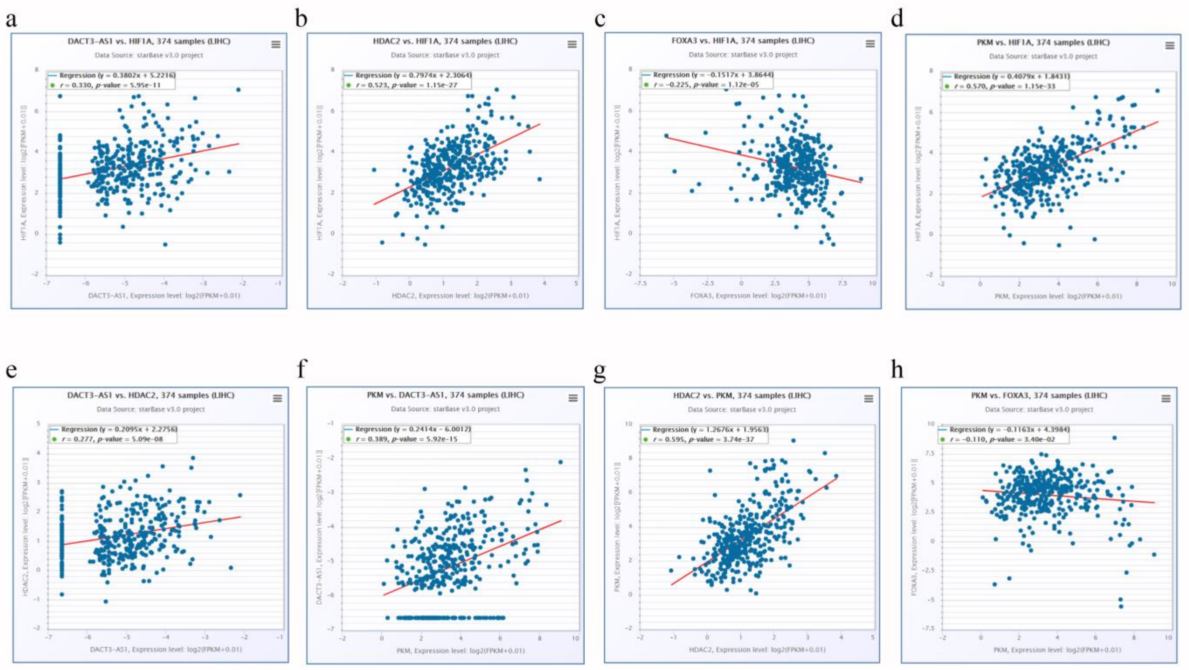

(a-d) The expression correlation between HIF-1α and DACT3-AS1/HDAC2/FOXA3/PKM2 in LIHC tissues was searched on starBase. (e-f) StarBase was applied to uncover the relationship between DACT3-AS1 and HDAC2/PKM2 expression in LIHC tissues. (g-h) Co-expression analysis for PKM2 and HDAC2/FOXA3 in LIHC tissues was achieved by means of starBase.

Graphical abstract

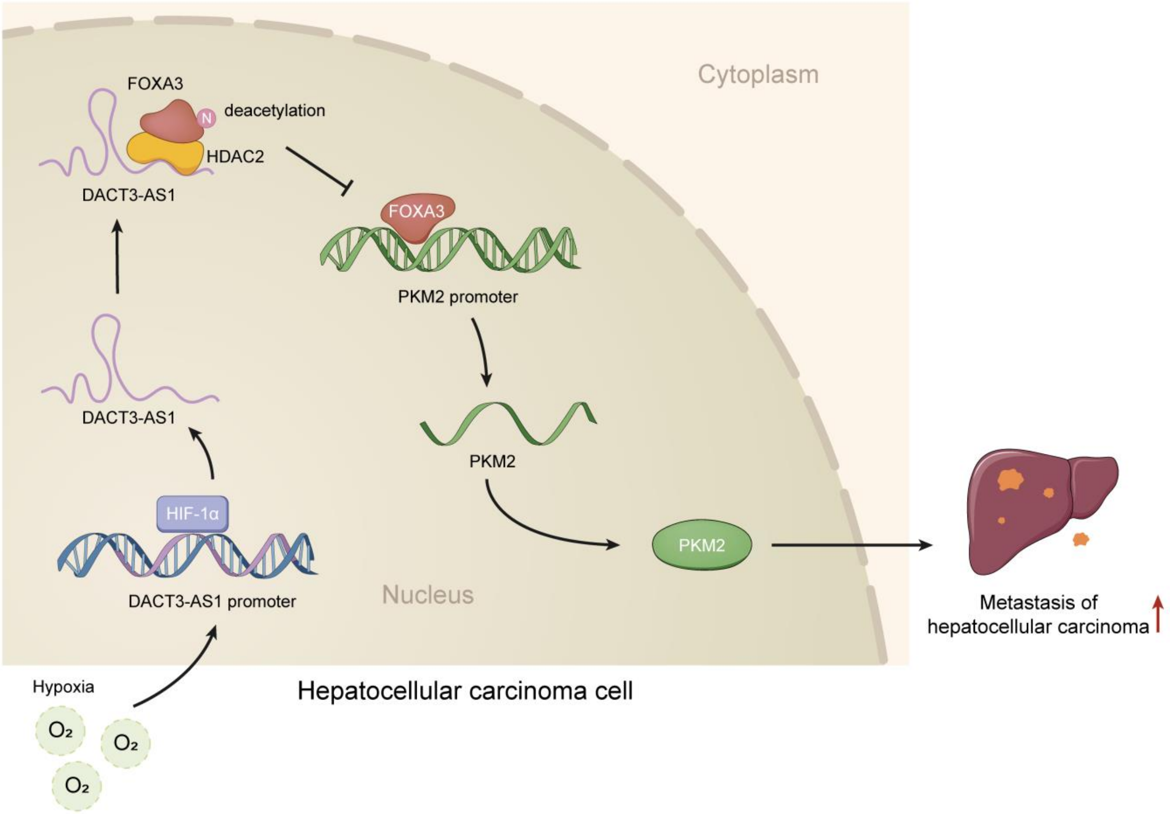

Supplement: Supplementary file 1 — Supplementary information [file 12276_2022_767_MOESM1_ESM.pdf]
